# Impact of Various Washing Protocols on the Mitigation of *Escherichia coli* Contamination in Raw Salad Vegetables

**DOI:** 10.3390/microorganisms12102103

**Published:** 2024-10-21

**Authors:** Fahad M. Alreshoodi, Bassam Alsuliman, Norah M. Alotaibi, Afnan Althobaiti, Lenah E. Mukhtar, Sarah Alsaleh, Abdullah A. Alajlan, Saleh I. Alakeel, Fahad M. Alshabrmi, Tarique Sarwar, Sulaiman M. Alajel

**Affiliations:** 1 Saudi Food and Drug Authority (SFDA), Riyadh 13513, Saudi Arabia; fmreshoodi@sfda.gov.sa (F.M.A.); bassamalsuliman@gmail.com (B.A.); nmaotaibi@sfda.gov.sa (N.M.A.); afnan.althob@gmail.com (A.A.); lemukhtar@sfda.gov.sa (L.E.M.); sarah.alsaleh@my.liu.edu (S.A.); aaajlan2@sfda.gov.sa (A.A.A.); siakeel@sfda.gov.sa (S.I.A.); 2 Department of Medical Laboratories, College of Applied Medical Sciences, Qassim University, Buraydah 51452, Saudi Arabia; t.sarwar@qu.edu.sa

**Keywords:** vegetable washing methods, salad vegetables, microbial contamination, *E. coli*, coliform, vinegar solution, consumer practices, food safety, ready-to-eat food, foodborne pathogens, infection, outbreak

## Abstract

Vegetables are an essential component of a balanced diet. The consumption of ready-to-eat foods may lead to the risk of infections and illnesses due to microbial contamination. To mitigate the potential of microbial contamination risks, it is critical to promote safe handling practices among consumers. In this study, our research evaluated the efficacy of different vegetable washing methods, specifically with lettuce, tomato, and cucumber, to establish optimal practices for reducing microbial contamination. This study consisted of two phases. Initially, a survey was distributed to 150 volunteers using snowball sampling to assess everyday vegetable handling and washing methods. The survey’s results identified four predominant methods: washing with a 5% vinegar solution for 3 min followed by tap water rinse (37.3% of participants), rinsing with tap water for 1 min (29.3%), washing with a 5% salt solution (vegetable soap) for 3 min followed by a tap water rinse (16.6%), and a 3 min tap water rinse (14%). A minor segment (3.33%) reported not washing their vegetables at all. The survey’s findings guided the second phase, which tested the aforementioned washing protocols’ effectiveness in reducing *Escherichia coli* (*E. coli*) levels on spiked contaminated salad vegetables. The tested vegetables were sterilized using UV light, inoculated with 0.5 McFarland *E. coli*, and then washed using the four identified methods. After that, *E. coli* enumeration after washing was performed using 3M™ Petrifilm and the comparison was analyzed via one-way ANOVA. During this study, it was revealed that the cucumbers had the highest *E. coli* contamination levels in comparison to the lettuce and tomato after washing. Interestingly, by comparing the three washing methods, it was found that washing the vegetables with vinegar proved to be the most effective solution for reducing microbial presence on both lettuce and cucumbers. Notably, the natural smoothness of tomato skin led to no significant differences in contamination levels across washing methods. In summary, vinegar washing effectively reduces microbial contamination from salad vegetables, highlighting the need for informed consumer practices to prevent foodborne outbreaks. This study emphasizes the importance of monitoring contamination sources and using safe washing techniques.

## 1. Introduction

Vegetables are essential to a nutritious diet, providing vital vitamins, antioxidants, dietary fibers, and minerals that enhance overall health. Global nutritional standards, including the [1], recommend a daily intake of at least five servings of vegetables due to their health benefits [2]. Despite this, the consumption of salad vegetables like lettuce, cucumbers, and tomatoes is commonly associated with microbial risks due to factors such as their sizeable surface-to-weight ratio and their slightly alkaline pH, which can encourage bacterial growth [3]. Common contaminants include pathogens, such as *Listeria monocytogenes*, *Salmonella*, and *Escherichia coli* O157:H7, which are often introduced to these vegetables and cause contamination at either the cultivation or the processing stages [4].

*Escherichia coli*, a prevalent pathogen linked to foodborne illness, contaminates fresh produce via multiple routes, with environmental conditions like temperature, pH, and humidity fostering microbial growth in vegetables [5,6,7]. Despite efforts to maintain low pathogen prevalence, the connection between ready-to-eat (RTE) food and foodborne illness outbreaks, especially those caused by *E. coli* O157:H7, remains a concern [8]. RTE foods are more susceptible to microbial contamination due to several factors. First, RTE foods are not cooked or reheated before consumption, which means bacterial contamination is not eliminated by heat. Second, RTE foods often undergo multiple stages of handling during preparation and packaging, thus increasing the chance of microbial cross-contamination. Third, RTE food storage conditions may not be sufficient to inhibit bacterial growth. Fourth, RTE foods have extended shelf lives, providing more time for bacteria to multiply if they are present. In addition, consumers may not handle RTE foods with the same care as raw ingredients, such as maintaining proper refrigeration or avoiding cross-contamination at home [9]. As global outbreaks linked to *E. coli*-contaminated vegetables have increased and consumer preferences have shifted towards greener diets, the risk of illness from pathogen-contaminated vegetables affects a broader population, particularly the vulnerable [10,11]. 

Notably, *E. coli* strains like *O157* can cause severe gastrointestinal symptoms, such as abdominal cramps and bloody diarrhea, as well as potentially fatal illnesses, such as hemolytic uremic syndrome (HUS) [12]. According to research, *E. coli O157* is highly widespread in undercooked meat and leafy greens, posing significant risks for common diets. This disease has a significant epidemiological impact, especially on vulnerable populations, including young children and the elderly, who are more likely to develop severe infections [10]. Furthermore, studies show that eating habits can increase the risk of O157 infections due to increased consumption of raw vegetables that may not be sufficiently well washed. The Enterobacteriaceae family is linked to a variety of foodborne illnesses, with some age groups being more susceptible, emphasizing the importance of customized food safety efforts [13]. The combination of these findings emphasizes the importance of our study and its implications for public health.

Investigating the most effective vegetable cleaning methods is critical for a variety of reasons. Cleaning vegetables thoroughly lowers the risk of contracting foodborne illnesses from pathogens, pesticides, and soil contaminants [14]. Furthermore, by eliminating toxic residues and preserving the produce’s inherent qualities, efficient cleaning maintains the nutritional value of the food [15]. Improving food safety procedures increases customer trust, which is essential for the public’s health. Additionally, knowing which cleaning techniques work best encourages environmentally friendly habits that cut down on water consumption and dangerous chemical usage. Identifying optimal procedures for processing ready-to-eat food can result in extended shelf life and improved visual appeal, boosting its marketability and reducing food waste [16]. Therefore, in order to advance food practices that are safer, healthier, and more sustainable, extensive research into cleaning techniques is essential. Although there are studies assessing the efficacy of various cleaning agents, no existing research compares cleaning techniques based on vegetable surfaces. 

Despite the increasing popularity of ready-to-eat vegetables, our study reveals a significant gap in consumer knowledge regarding the role they play in reducing foodborne illnesses and the effectiveness of household vegetable-washing methods in regions like Saudi Arabia, where raw vegetable consumption is prevalent. Consumers need to be educated about proper sanitation methods to mitigate risks, especially since the consumption phase is the final step in the ‘farm to fork’ process and falls outside the scope of regulatory inspections. Therefore, this study aims to assess consumer sanitation practices and investigate the efficacy of various washing procedures (e.g., tap water and natural sanitizers such as vinegar, and vegetable detergents) in reducing *E. coli* on salad vegetables. Our evidence-based recommendations can significantly improve food safety practices and help prevent foodborne outbreaks.

## 2. Materials and Methods

### 2.1. Study Design

This investigation was conducted in two phases to enhance our understanding of raw vegetable hygiene practices and their impact on *E. coli* contamination reduction. The first phase involved a structured survey to gather comprehensive data on consumers’ handling and washing practices when dealing with raw vegetables. This phase was crucial in identifying prevalent behaviors and strategies used in consumer kitchens, which informed the design of the second phase. Leveraging the findings from the first phase, the second phase was meticulously designed to assess the effectiveness of real-world vegetable management techniques. This phase utilized controlled experiments to simulate the handling and washing practices commonly employed by the public, evaluating their efficacy in minimizing *E. coli* contamination on raw vegetables. The objective was to bridge the gap between consumer habits and scientific validation of their effectiveness in enhancing food safety.

### 2.2. Survey on Domestic Raw Vegetable Washing

A questionnaire was disseminated to a voluntary sample of 150 participants chosen through snowball sampling methodology [17]. The questionnaire was specifically crafted to elicit insights into the practices of vegetable sanitation within domestic environments. All participating subjects responded to the questions, totaling four, which can be made available upon request. The survey aimed to capture data related to the techniques utilized for washing vegetables in household contexts, emphasizing methods like immersion, running water, and additives introduced to the washing process.

### 2.3. Washing Protocols

The investigation identified three distinct washing procedures employed in domestic settings to clean and sanitize raw vegetables. These methodologies were subsequently analyzed in a controlled experimental framework to determine their effectiveness in mitigating or completely removing *E. coli*, which were deliberately inoculated on the vegetable samples. The washing protocols under investigation were delineated as follows:

Washing Protocol 1 (WP1). This protocol involved a 3-min immersion of fresh vegetables in a 5% vinegar solution (the company was not disclosed as it was provided by the government) followed by a 3-min rinse under tap water [18]. 

Washing Protocol 2 (WP2). This approach entailed a 3-min immersion in a 5% salt solution (vegetable soap—the company was not disclosed as it was provided by the government) followed by a 3-min rinse with tap water.

Washing Protocol 3 (WP3). This procedure included a 3-min rinse under tap water.

### 2.4. Sample Collection

Lettuce, cucumber, and tomato vegetable samples were randomly sourced from Riyadh, Saudi Arabia, retailed between March and December 2022. Any visibly deteriorated portions were carefully excised from these samples. After that, the vegetables were preserved at a refrigeration temperature of 4 °C pending further processing. For experimental preparation, 10 g from each vegetable sample underwent an initial cleansing phase consisting of a 10 s surface rinse with 70% methanol followed by tap water. Vegetable samples were sterilized using UV radiation in a biosafety cabinet for 30 min (15 min per side, 36,000 µJ/cm^2^) to minimize native microflora and initial microbial load [19]. 

### 2.5. Bacterial Strain and Inoculation Procedure

This research investigates eleven *E. coli* O157 isolates, consisting of ten samples obtained from the Biobank reference lab of microbiology at the Saudi Food and Drug Authority and one sample from ATCC 43888, all derived from food products. Identification of these samples was conducted according to ISO standardization methods before their final preservation in the biobank. The indole test was employed as part of the identification process to confirm the presence of *E. coli*. To revive the cultures, the bacterial isolates were inoculated onto Sorbitol-MacConkey agar (Oxoid, Hampshire, UK) and incubated at 37 °C for 24 h, facilitating accurate growth and enabling further characterization of the isolates. A 0.5 McFarland bacterial suspension was prepared and subsequently diluted by transferring 1 mL of the suspension into a separate tube containing saline (Merck, Darmstadt, Germany). To spike the samples, 100 µL of the diluted bacterial suspension was taken, spread evenly onto the sample surface and allowed to air dry for one hour.

### 2.6. Microbiological Analysis

Following the decontamination of the vegetable samples, a rigorous microbiological analysis was undertaken to quantify bacterial presence. A 10 g portion of each vegetable sample was placed in stomacher bags and subjected to a 1:10 dilution with buffered peptone water (BPW) (Oxiod, Hampshier, UK). This was followed by homogenization at standard speed utilizing a stomacher apparatus (Biomatyx, Avelin, France) (International Organization for Standardization 16654, 2023). In adherence to the ISO 16654 standard [20], aliquots of the homogenized samples were plated in triplicate onto 3M™ Petrifilm™ (3M Science, St. Paul, MN, USA) and incubated at 37 °C for 24 ± 4 h. Post-incubation, the bacterial colonies were quantified, and the results were expressed as the logarithmic value of colony-forming units per gram of vegetable (log CFU/g).

### 2.7. Statistical Analysis

This study’s graphical representations and statistical evaluations were generated and performed employing GraphPad Prism software version 9.0 (GraphPad Software, La Jolla, CA, USA) based on the data derived from three independent biological experiments. Before the analysis, bacterial counts were logarithmically transformed to compute means and standard deviations, with the growth of bacteria being expressed as log CFU/g. A two-tailed unpaired *t*-test with a 95% confidence interval was utilized to assess statistical significance in pairwise comparisons. A one-way Analysis of Variance (ANOVA) was conducted to evaluate statistical distinctions across multiple treatment groups with a 95% confidence interval, incorporating Tukey’s multiple comparisons test as a post hoc correction. A *p*-value of less than 0.05 was deemed indicative of statistical significance.

## 3. Results

### 3.1. Questionnaire Data

In the preliminary phase of this research, a questionnaire was disseminated among 150 volunteers, who were recruited via a snowball sampling technique, to collate information on the standard practices employed in handling and washing raw vegetables domestically. Analysis of the questionnaire responses revealed four predominant vegetable cleansing methods utilized by the participants. Approximately 37.3% of the respondents described a regimen wherein vegetables were immersed in a 5% acetic acid (vinegar) solution for three minutes, followed by a rinsing process with potable water (WP1). About 29.3% of the surveyed participants reported that they exposed their vegetables to a rinse under flowing tap water for one minute (WP2). Around 16.6% of the individuals involved in this study employed a method of vegetable immersion in a 5% sodium chloride (salt) solution for three minutes, followed by a rinse in tap water (WP3). Close to 14% of respondents preferred to rinse their vegetables under tap water for three minutes (WP4). A small subject, 3.33%, of the surveyed population reported abstaining from vegetable-washing practices (Figure S1).

### 3.2. Measuring the Microbial Loads

**Figure 1:**
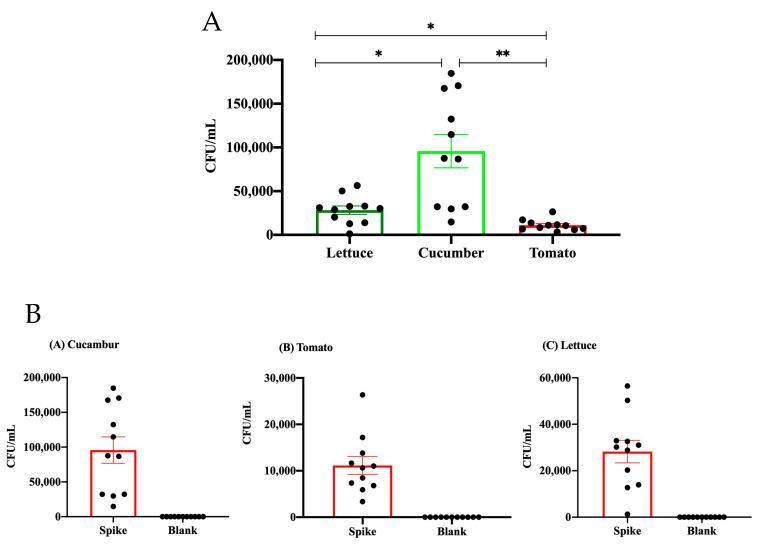
(**A**) A visual representation of the relative microbial load of E. coli strains observed in lettuce, cucumber, and tomato. Bars are means ± SEM. Data were analyzed using one-way ANOVA (* *p* < 0.05, ** *p* < 0.01). (**B**) A figure representing a comparison of microbial growth between the spiked and blank sterilized samples. The *p*-values for the tested vegetables are less than 0.0001.

The microbial load was quantified as colony-forming units per milliliter (CFUs/mL) across 11 different foodborne *E. coli* O157 isolates for each treatment (Figure 1A). During spiking the samples with the bacteria, it was found that the lettuce samples had relatively low CFU/mL values, suggesting a concentrated distribution and little variability in contamination levels. In comparison to lettuce and tomato, cucumbers demonstrated notably higher CFU/mL values, indicating a significant variability in microbial load. Among the three types of vegetables, tomatoes exhibited the lowest microbial load, as evidenced by the closely grouped data points located towards the lower end of the y-axis. This clustering suggests a continuous and sustained presence of contamination at low levels. The statistical study depicted in Figure 1A reveals significant differences in microbial load among the different kinds of produce, with cucumbers consistently exhibiting the most elevated levels of contamination.

Next, the microbial load in the tested spiked samples was compared to the blank ones (Figure 1B). Among the spiked samples, cucumbers exhibited the greatest microbial load, characterized by a substantial dispersion of data points, suggesting notable variability. The spiked tomato samples had a modest microbial burden, as seen by data points demonstrating reduced variability in comparison to cucumbers. The spiked samples of lettuce had a greater microbial load, while the data points displayed a more organized clustering pattern, suggesting a higher degree of consistency in contamination levels. In all three produce types, the blank samples showed negligible microbial loads, confirming the effectiveness of the spiking procedure in this experiment.

### 3.3. Comparison of Washing Methods

**Figure 2:**
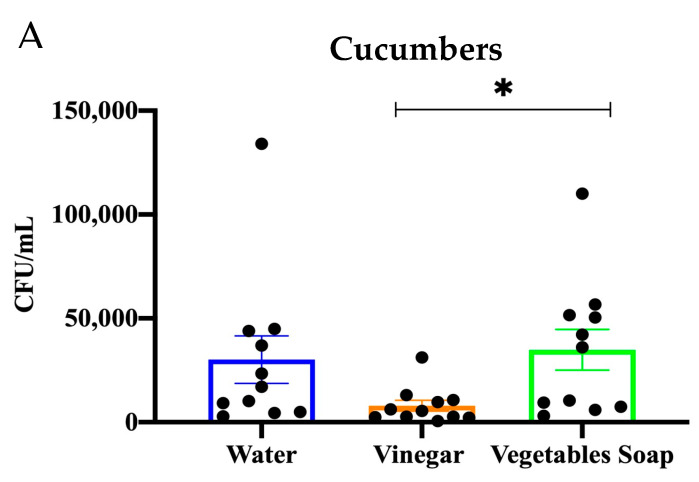

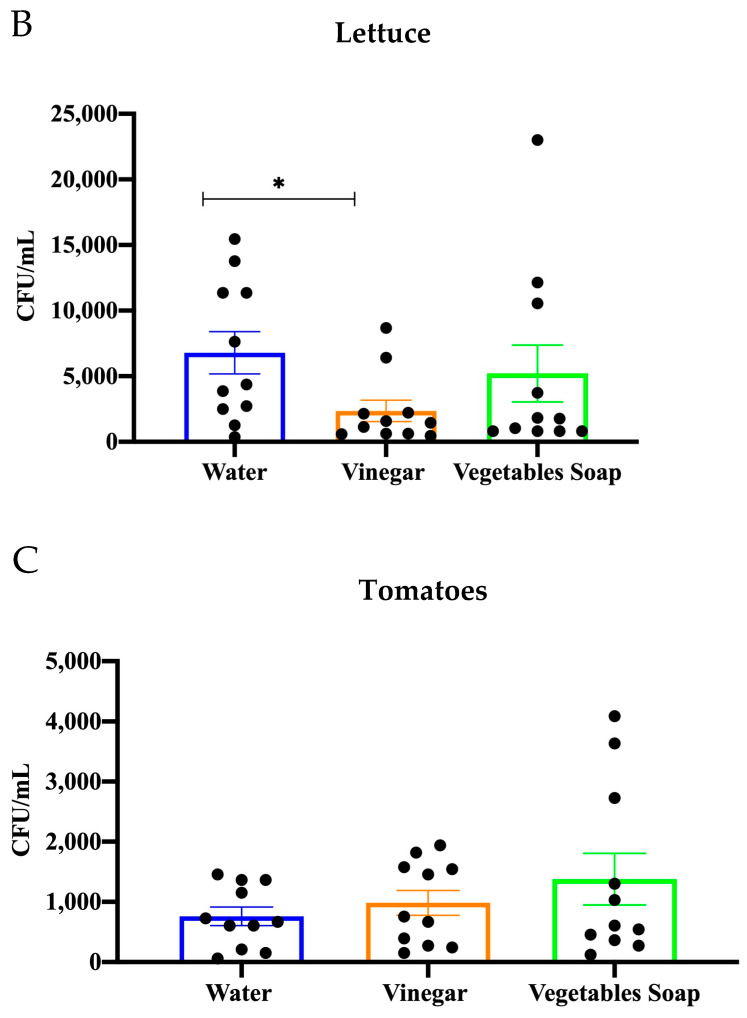
(**A**) A bar graph shows the effectiveness of three washing methods on the cucumber surface. Bars are means ± SEM. Data were analyzed using one-way ANOVA (* *p* < 0.05). (**B**) A bar graph demonstrating *E. coli* growth in lettuce after using the washing methods. Bars are means ± SEM. Data were analyzed using one-way ANOVA (* *p* < 0.05). (**C**) A bar graph demonstrating *E. coli* growth in tomatoes after using the washing methods. Bars are means ± SEM. Data were analyzed using one-way ANOVA.

**Figure 3:**
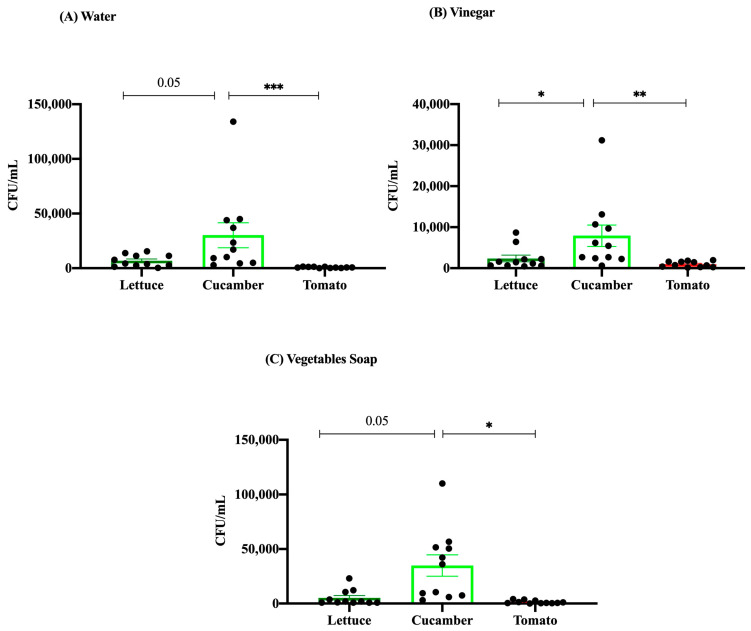
Effect of (**A**) water, (**B**) vinegar, (**C**) and vegetable soap on vegetable surfaces. No effects of cleaning methods were found on cucumber. Bars are means ± SEM. Data were analyzed using the Friedman test (* *p* < 0.05, ** *p* < 0.01, *** *p* < 0.001).

The efficacy of diverse decontamination strategies on three types of vegetables (tomato, lettuce, and cucumber) was evaluated to determine their ability to reduce bacterial contamination by employing distinct washing methods—namely, deionized water, an acetic acid (vinegar) solution, and a salty vegetable cleanser (Figure 2 and Figure 3). 

#### 3.3.1. Cucumber

For the cucumber, the effectiveness of three washing treatments, including water, vinegar, and vegetable soap, in lowering microbial load of the *E. coli* was assessed by quantifying the CFU/mL on the surfaces of the product after the treatment process (Figure 2A). There was considerable variation in the microbial load seen among the various treatments investigated.

The cucumbers that underwent water washing demonstrated the most elevated levels of microbial burdens, with CFU/mL values spanning a broad range from almost negligible to about 150,000, where the average CFU/mL for water-treated samples was significantly greater than that of the other treatments, suggesting a restricted efficacy in diminishing microbial contamination.

Conversely, the application of vinegar treatment yielded a notable decrease in microbial load, as seen by most samples exhibiting CFU/mL values that approached zero. Vinegar-treated samples had the lowest mean CFU/mL compared to the other two groups, indicating the higher effectiveness of vinegar in decontaminating microbial organisms.

For the vegetable soap, the results showed a decrease in the microbial load, as seen by mean CFU/mL values that were lower compared to water-treated samples; however, they were greater than those treated with vinegar. However, while vegetable soap showed more efficacy compared to water, it did not attain the same degree of microbial reduction as vinegar.

The findings of the present investigation on the cucumbers indicate that vinegar has superior efficacy as a washing agent in mitigating microbial contamination on vegetables, surpassing the effectiveness of both water and vegetable soap.

The analysis of the washing methods revealed a statistically significant impact on the decontamination efficacy for cucumbers, as indicated by a *p*-value of 0.0093 F = (7.762). Multiple comparison tests were employed to investigate the treatment groups’ distinctions further. The results demonstrate a statistically significant difference in the bacterial reduction efficacy between the vinegar solution and the vegetable soap treatments (*p* = 0.017). Conversely, there was no statistically significant difference observed in comparing bacterial reduction between the water and vinegar treatments (*p* = 0.09) or between the water and vegetable soap treatments (*p* = 0.6).

#### 3.3.2. Lettuce

The washing methods showed a significant effect on lettuce, with F = (4.389) and *p* = 0.0361. The microbial load on the samples treated with water exhibited moderate levels, with ranges of CFU/mL values spanning from negligible to over 20,000. The average CFU/mL for samples treated with water a significant increase in comparison to the other treatments, suggesting a comparatively limited effectiveness in mitigating microbial contamination. Conversely, the application of vinegar treatment yielded a significant decrease in microbial burden (*p* = 0.01), as seen by the majority of samples exhibiting CFU/mL values far below 5000. Similarly, with cucumbers, the vinegar-treated samples exhibited the lowest mean CFU/mL compared to the other two groups, indicating vinegar’s higher efficacy in reducing microbial growth. On the other hand, the application of vegetable soap treatment also led to a decrease in microbial load, while the average CFU/mL was higher compared to the vinegar but lower than that found with water (*p* = 0.1). This finding suggests that vegetable soap exhibits greater efficacy compared to water, although it demonstrates lower efficacy in mitigating microbial contamination when compared to vinegar. In conclusion, these findings suggest that vinegar exhibits superior efficacy in mitigating microbial load on vegetables, surpassing the effectiveness of both water and vegetable soap. 

#### 3.3.3. Tomato

The testing of the washing method on the tomato was analyzed using the ANOVA test; F = (1.162) and *p* = 0.3265. The multiple comparisons showed no significance between groups. The water-washed samples exhibit a moderate range of microbial burdens, with CFU/mL levels generally falling below 2000. The average CFU/mL observed in the water-treated group suggests that water alone offers an adequate level of microbial reduction, while it does not effectively eliminate the contamination.

In comparison to the water-treated group, the samples treated with vinegar demonstrated a slight reduction in microbial burdens (*p* = 0.7), as shown by CFU/mL values that tended to cluster around 1000. The average CFU/mL for samples treated with vinegar was found to be identical to that of samples treated with water, indicating that vinegar exhibited a comparable degree of microbial decrease in this particular case.

On the other hand, the application of vegetable soap treatment led to a greater degree of variability in the microbial load (*p* = 0.6), as certain samples had elevated CFU/mL levels surpassing 3000. The average CFU/mL for this group treated with vegetable soap was found to be greater compared to both the water and vinegar groups. This suggests that vegetable soap exhibited less consistency and effectiveness in lowering microbial burdens in the specific context under investigation.

In contrast to the previous data, the current figure does not demonstrate a statistically significant difference among the three treatments. This finding indicates that, in the present scenario, none of the treatments yielded a statistically significant decrease in microbial burden.

Overall, the present study aimed to assess the efficacy of three distinct washing treatments, including water, vinegar, and vegetable soap, in reducing the microbial load of three different types of produce, namely, lettuce, cucumber, and tomato. The findings are delineated into three distinct panels, namely, panel A for water, panel B for vinegar, and panel C for vegetable soap (Figure 3).

Upon washing the produce with water (panel A), a notable disparity in microbial load was noted among the various varieties of vegetables. Cucumber exhibited the highest microbial load, with CFU/mL values reaching a maximum of 100,000 individual units. In contrast, lettuce and tomato demonstrated significantly reduced microbial burdens, as shown by CFU/mL values that were generally in close proximity to zero. The results of the statistical analysis indicated a statistically significant disparity in microbial load between cucumber and both lettuce and tomato (*p* < 0.05).

With respect to panel B, the implementation of vinegar as a cleansing agent resulted in a notable decrease in the microbial burden observed in various types of produce. Nevertheless, cucumber demonstrated the largest microbial load, but at far lower levels compared to those detected during water treatment, with values reaching a maximum of approximately 30,000 CFU/mL. Lettuce and tomato exhibited reduced microbial burdens, with values markedly lower than those of cucumber. The statistical study provided support for the presence of substantial differences between cucumber and both lettuce and tomato (*p* < 0.05 and *p* < 0.01, respectively).

In terms of panel C, the application of vegetable soap treatment also led to a decrease in microbial load, exhibiting comparable patterns to those reported in the water treatment analysis. Cucumber once again demonstrated the highest microbial load, displaying CFU/mL levels that were similar to those observed in the water treatment, reaching in excess of 100,000. The microbial loads of lettuce and tomato were found to be much lower, with the majority of the results approaching zero. This study revealed a statistically significant disparity in the microbial loads between cucumber and the other two types of vegetables (*p* < 0.05).

In summary, it can be observed that cucumber consistently displayed larger microbial loads in comparison to lettuce and tomato across all experimental washing regimes. Among the three treatments examined, vinegar showed the highest efficacy in mitigating the microbial load, namely, in the case of cucumber. However, it is important to note that vinegar did not entirely eradicate microbial contamination. The efficacy of water and vegetable soap treatments was found to be slightly lower since vegetable soap exhibited a comparable decrease in microbial load to that of water. Prominent disparities in microbial load were routinely noted between cucumber and the remaining two types of vegetables, underscoring the variability in the effectiveness of washing treatments based on the specific produce variety. The aforementioned findings highlight the significance of carefully choosing suitable washing methods in order to efficiently mitigate microbial contamination on various categories of agricultural products.

## 4. Discussion

This comprehensive study explores the efficiency of commonly employed washing techniques to mitigate *E. coli* contamination from salad vegetables. Given the potential for microbial contamination in raw salad vegetables, such as lettuce, cucumber, and tomato, to precipitate significant health outbreaks, understanding and disseminating effective decontamination practices is crucial. This concern is underscored by data from the Center for Disease Control and Prevention (CDC), which explicitly links numerous recent *E. coli* outbreaks to vegetable consumption, particularly leafy greens like lettuce [21]. Hence, identifying optimal consumer practices for eliminating pathogenic bacteria from vegetables is pivotal in averting potential bacterial outbreaks.

Our investigation reveals a diversity in consumer approaches to vegetable disinfection, with a notable preference for immersing vegetables in a 5% white vinegar solution followed by a rinse in tap water, as depicted in Figure 3. Alternative methods reported include the use of vegetable soaps, lemon, or apple cider vinegar, highlighting a spectrum of practices among consumers [18]. This variation underpins the necessity for clear guidance on the most effective and safe vegetable-washing practices. Echoing the U.S. Food and Drug Administration’s recommendations, the ideal method involves scrubbing vegetables under running tap water without using specialized vegetable detergents, which currently lack comprehensive effectiveness studies [22]. 

Through the lens of this research, we meticulously assessed the impact of three distinct washing procedures—water only, vinegar, and specialized vegetable soap—on *E. coli* contamination in lettuce, tomatoes, and cucumbers. Our results demonstrate vinegar’s efficacy in reducing *E. coli* levels, particularly in lettuce and cucumbers. This outcome likely stems from vinegar’s antimicrobial properties, primarily attributed to its acetic acid content, corroborating findings from prior research [23,24]. Its mode of action includes numerous critical mechanisms. First, vinegar considerably decreases the pH of the vegetable surface, resulting in an acidic environment that prevents the growth of numerous bacteria, molds, and pathogens [25]. Acetic acid can kill microbial cells by penetrating their membranes. It also inhibits vital enzymatic functions in bacteria, impeding their metabolic processes. Furthermore, vinegar serves as a solvent, efficiently dissolving biofilms and residues that may contain pollutants [26]. Interestingly, commercial vegetable soap outperformed lettuce by mere water, suggesting that while soaps may dislodge bacteria from the vegetable’s surface, they do not inherently reduce microbial loads more effectively than water. 

Water is required for vegetable washing and acts via a variety of methods. For instance, it makes it easier to physically remove dirt and debris off vegetable surfaces since rinsing or soaking helps pollutants to be loosened and rinsed away. Water can also permeate the porous surfaces of certain plants, hydrating and loosening any particles that have attached to them. It also helps to dissolve water-soluble pollutants, such as insecticides, so they can be readily rinsed away. Water’s reduced surface tension allows it to flow more effectively across the vegetable’s surface, penetrating into crevices where dirt and bacteria can lurk. Using warm water can also improve cleaning efficiency by increasing the solubility of some contaminants [27]. 

In cucumbers, there was no significant disparity observed between water and vegetable soap, indicating a potential lack of antimicrobial efficacy in soaps for bacteria firmly adhered to the cucumber’s surface. Some commercial vegetable soaps produce similar outcomes to water while cleaning vegetables for a variety of reasons. First, if the contaminants are mostly water soluble, such as pesticides or soil, both water and soap can effectively remove them, yielding similar results. Furthermore, a soap’s efficiency is determined by its formulation; if it lacks powerful surfactants or antibacterial ingredients, it may operate similarly to water. Smooth-skinned vegetables may not benefit as much from soap as those with more complicated surfaces. Furthermore, if the microbial load on the vegetables is modest, the difference in efficacy between soap and water may be minimal. However, vinegar’s pronounced bacterial count reduction in cucumber surfaces underscores its potency. The efficacy of vegetable cleaning procedures might vary greatly depending on the type of vegetable being cleaned. For example, leafy greens like lettuce have a complex surface with fissures that can trap dirt and germs, making them more difficult to clean than smooth-skinned plants [28,29]. Furthermore, some plants, such as cucumbers, have a natural waxy layer that can retain residues; in such circumstances, a vinegar wash may aid in the removal of these contaminants [30]. Certain vegetables, such as mushrooms, have high porosity, which makes cleaning more difficult because their surfaces can absorb water and pollutants. Furthermore, microbial contamination levels might vary amongst vegetable kinds due to changes in the growing environment, handling, and storage procedures [31]. 

In addition, the use of vinegar in washing vegetables recently raised concerns about the potential emergence of acid-resistant pathogenic strains capable of surviving human gastrointestinal conditions [32,33]. This highlights the need for moderation and further research to optimize organic acid concentrations for safe and efficient produce disinfection. 

Furthermore, the smooth skin of tomatoes likely facilitates easier bacterial removal compared to the rougher surfaces of lettuce and cucumbers, which provide more tenacious bacterial adherence points. Consistently, our data revealed no significant differences among the washing methods for tomatoes, whereas lettuce and cucumbers showed a distinct need for antimicrobial agents, like vinegar, for substantial bacterial reduction. This conclusion is consistent with the findings of Leff et al., 2013, which indicate that fruits and vegetables host varied bacterial communities depending on their structure [34]. 

In conclusion, this research highlights the importance of specific cleaning processes based on the type of vegetable, as different plants may harbor various kinds and quantities of contamination. Cleaning procedures customized for certain vegetable types can improve food safety and reduce the possibility of microbial contamination, resulting in improved health ends for customers. We also need more studies to determine the best cleaning agent concentrations and to perform investigations that evaluate bacterial processes in response to acids. Further studies will allow us to better understand how varying concentrations influence microbial behavior and effectiveness.

Given the public health implications, governmental bodies must promote safe food handling practices and issue guidelines to mitigate the risk of pathogenic bacteria outbreaks, safeguarding community health and well-being.

## 5. Conclusions

The findings from our investigation indicate that the three utilized washing techniques significantly impact the reduction in microbial presence on vegetable surfaces. Consumers must adopt effective disinfection practices for their food products to mitigate the risk of foodborne pathogens and subsequent bacterial outbreaks. Our research underscores the antimicrobial potential of vinegar, particularly its efficacy in decreasing *Escherichia coli* populations on the surfaces of salad vegetables, with an enhanced effect observed in leafy vegetables, such as lettuce. Additionally, our study corroborates the concept that vegetable morphological characteristics—specifically, the texture and structure of their surfaces—play a crucial role in bacterial adhesion and, consequently, influence the success of different washing methods. This observation underscores the importance of considering vegetable morphology when evaluating or recommending cleaning practices. Given these insights, there is an apparent necessity for widespread public education on safe and effective vegetable cleaning techniques. Targeted advertising campaigns and informational initiatives within grocery stores, supermarkets, and local vegetable shops could substantially enhance consumer awareness and knowledge. Such efforts should provide clear, research-backed guidance on the best practices for reducing the risk of contamination and ensuring the safety of produce consumed by the public.

## Data Availability

The original contributions presented in the study are included in the article/Supplementary Materials, further inquiries can be directed to the corresponding authors.

## Supplementary Materials

The following supporting information can be downloaded at: https://www.mdpi.com/article/10.3390/microorganisms12102103/s1, Figure S1: A graph demonstrated the results of the survey of the domestic washing methods used on the leafy green vegetables. Most of the participants wash the vegetables using 3 min immersion in a 5% solution of vinegar in water, followed by a tap water rinse.
