# Supplementary material for: Impact of Various Washing Protocols on the Mitigation of Escherichia coli Contamination in Raw Salad Vegetables
Source: Microorganisms. 2024 Oct 21;12(10):2103. doi: 10.3390/microorganisms12102103 (PMC11510425; doi:10.3390/microorganisms12102103)
Supplement: Supplementary file 1 [file microorganisms-12-02103-s001.zip › microorganisms-3216366-supplementary.pdf]

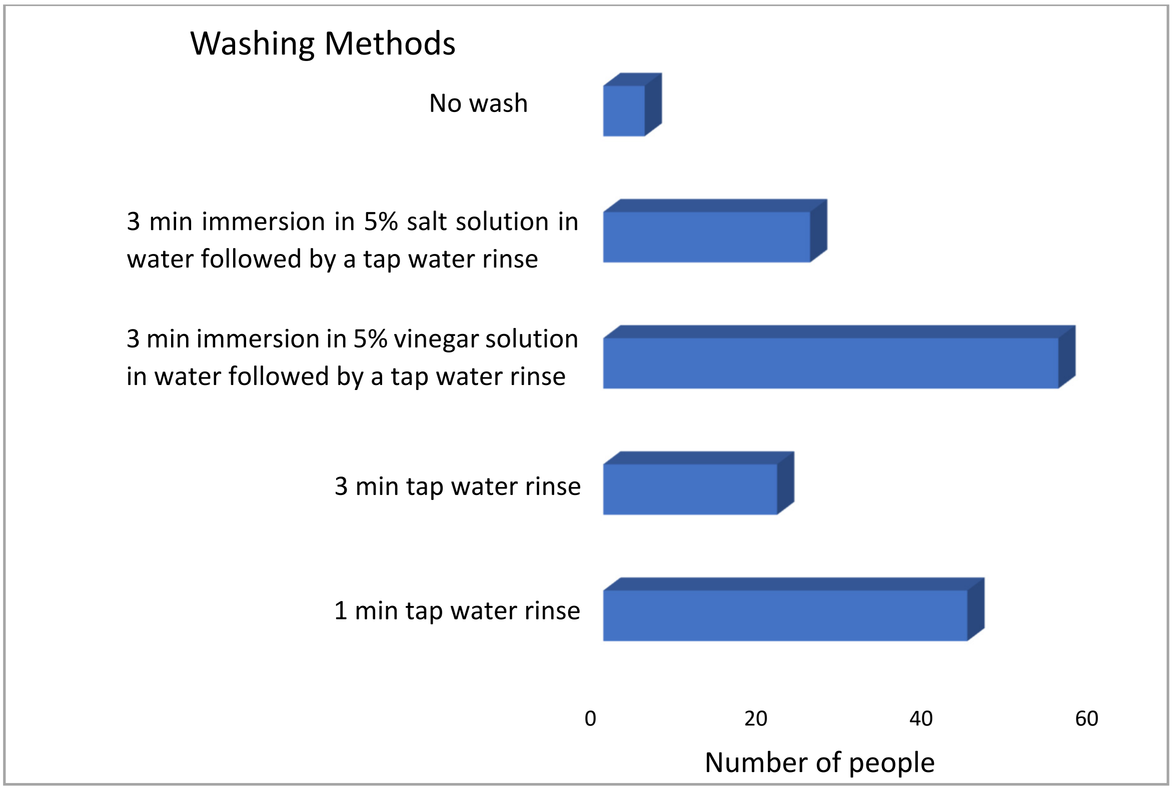

Figure S1. A graph demonstrated the results of the survey of the domestic washing methods used on the leafy green vegetables. Most of the participants wash the vegetables using 3 min immersion in a 5% solution of vinegar in water, followed by a tap water rinse.
